# The Role of Moral Foundations, Anticipated Guilt and Personal Responsibility in Predicting Anti-consumption for Environmental Reasons

**DOI:** 10.1007/s10551-021-05016-7

**Published:** 2022-01-08

**Authors:** Barbara Culiberg, Hichang Cho, Mateja Kos Koklic, Vesna Zabkar

**Affiliations:** 1 School of Economics and Business, University of Ljubljana, Kardeljeva ploscad 17, 1000 Ljubljana, Slovenia; 2 Department of Communications and New Media, National University of Singapore, 11 Computing Drive, AS6 03-13, Singapore, 117416 Singapore

**Keywords:** Moral foundations, Air travel, Anti-consumption, Environmental activism, Anticipated guilt, Personal responsibility, WOM

## Abstract

In response to the growing importance of environmental issues, more and more consumers are turning to anti-consumption by reducing, rejecting, or avoiding consumption. Covering the intersection of sustainable consumption and anti-consumption, previous studies relied on socio-cognitive models to explain this decision. In order to extend their findings, we consider the moral and emotional perspectives to examine reducing consumption for environmental reasons in a particular context, i.e. air travel. It is against this backdrop that we propose a conceptual model that includes moral foundations as the main antecedent, followed by anticipated guilt and personal responsibility, while intention to reduce consumption (i.e. air travel) for environmental reasons, positive word of mouth about reducing air travel (WOM) and environmental activism represent the outcomes. The proposed model is tested on a sample of 511 respondents from a UK online consumer panel. Our results confirm the importance of moral foundations, anticipated guilt and personal responsibility and their interplay in the prediction of intention to reduce consumption for environmental reasons. Anticipated guilt influences WOM, while personal responsibility influences activism. In addition, intentions to reduce consumption for environmental reasons have a positive impact on WOM and environmental activism. There are several implications for public policy makers and NGOs that fight against climate change that derive from these findings, as well as research opportunities for academics interested in this topic.

## Introduction

Climate change is one of the most serious issues concerning our planet today. Although the levels of CO_2_ emissions dropped in 2020 as the result of the COVID-19 pandemic (Le Quéré et al., 2020), climate change is not on pause, but the emissions are expected to return to higher levels after the pandemic has ended (UN, 2021). As concern for the environment is growing, people have started to curb their consumption in various areas of their lives. A recent survey on how individuals intend to fight climate change (European Investment Bank, EIB, 2021) reported that if given the choice to give up various goods or services, 40% of Europeans would find it easiest to give up flying (followed by video streaming, meat, clothes and cars). With the rise of the Swedish-born movement of flight-shaming or “flygskam”, supported by Greta Thunberg, it is not surprising that more and more people are considering reducing their flights in the future, and this trend is likely to continue in developed markets, especially as the COVID-19 pandemic has given more support to this cause (Berton, 2019; EIB, 2021; Gössling et al., 2019). Hence, in order to understand the drivers and outcomes of reducing consumption for environmental reasons, we focus on air travel, which is an appropriate study setting, especially considering the airline industry is one of the top 10 global emitters of CO_2_ and is expected to account for 25% of all such emissions by 2050 (UNFCC, 2020).

When studying consumers who reduce or restrict consumption *for environmental reasons*, we need to consider the intersection of the literature on sustainable consumption^1^ and anti-consumption (Scott & Weaver, 2018). In this domain we can also find environmental anti-consumption described “as acts directed against any form of consumption, with the specific aim of protecting the environment” (García-de-Frutos et al., 2018). The area of anti-consumption is relevant, because it deals with the reasons for not engaging in consumption, which are clearly different from those in favour of consumption (Chatzidakis & Lee, 2013). Anti-consumption requires meaningful lifestyle changes and comes in different forms, varying in terms of actors, goals, targets, duration and intensity (Chatzidakis & Lee, 2013; Sekhon & Armstrong Soule, 2020). The main focus of anti-consumption studies has been on reducing, avoiding and rejecting consumption (García-de-Frutos et al., 2018; Lee et al., 2020). Early studies in this field included topics such as voluntary simplicity (see Craig-Lees & Hill, 2002; Leonard-Barton, 1981; Shama, 1981), which was associated with reduced consumption lifestyles, and found that transport was one of the most contentious items on the research agenda (Shaw & Newholm, 2002). Later, some scholars examined anti-consumption in more general terms (Lee et al., 2009; Sekhon & Soule, 2019), while others provided insights into specific contexts and product categories (i.e. electronic goods, food and beverages, clothing and sports products) with the potential to expand these further afield (Makri et al., 2020). Accordingly, our study is set in a particular anti-consumption context, namely reducing air travel. More specifically, considering Iyer and Muncy’s (2009) typology of anti-consumption, which divides anti-consumption based on the object (general or a specific product, brand, or activity) and the purpose (individual or societal reasons), our work can be positioned under the domain of market activists, which represent individuals who refrain from a potentially harmful activity (e.g. air travel) because it causes a specific societal problem (e.g. climate change).

Previous studies at the crossroads of anti-consumption and sustainable consumption relied on socio-cognitive models and theories, two of the most popular being Ajzen’s (1991) Theory of Planned Behaviour (TPB) (e.g. Chen, 2016; Yarimoglu et al., 2019) and Stern et al.’s (1999) Value–Belief–Norm Theory (VBN) (e.g. Jakovcevic & Steg, 2013; Zhang et al., 2020), which primarily revolve around the normative- and reason-based approaches to explain the decision to pursue or avoid certain behaviours. The common denominators of these approaches are socio-psychological factors, such as beliefs, norms, values and attitudes which have been found to explain both anti-consumption and sustainable consumption (see Bamberg & Möser, 2007; García-de-Frutos et al., 2018). However, with the progression of these research fields alternative models and concepts, such as feelings (see White et al. 2019) and moral variables (e.g. Watkins et al., 2016) have been introduced, suggesting reason is not always at the heart of making such choices, thus paving the way for studies on anti-consumption for environmental reasons.

The research on anti-consumption is diverse and extensive, but on closer inspection we were able to identify under-investigated areas in the literature that have led us to pursue the matter further. The main premise behind environmental anti-consumption is that environmental resources are generally available to everyone, and therefore, the use of these resources by one consumer affects others, because it reduces the amount that is available. Through anti-consumption individuals may reduce their overall material quality of life, yet improve the situation of others, as the unused resources thus remain available for use (Kaiser & Shimoda, 1999). However, more recent studies have also shown that anti-consumption can actually enhance the well-being of those who practice it (see Hoffmann & Lee, 2016; Chowdhury, 2018). Regardless of these proposed benefits, when faced with a choice for or against behaviour which contributes to climate change, such as air travel, consumers are inevitably dealing with a moral dilemma, since they have to decide whether to engage in a behaviour that potentially damages the environment, or help save the environment by avoiding it. This regulation of self-interest (versus societal interest) is central to morality (Campbell & Winterich, 2018). It is therefore not surprising that morality has been identified as a relevant predictor in the domain of anti-consumption (Makri et al., 2020), and although this has previously remained on the sidelines of such discussions it has recently been moving to the forefront of scholars’ interest (see Azevedo, 2020; García-de-Frutos et al., 2018; Muncy & Iyer, 2021; Peifer et al., 2020; Sudbury-Riley & Kohlbacher, 2018). Sekhon and Soule (2019) identified a gap in the literature with regard to the role of contextually relevant consumer characteristics and the motives that people ascribe to anti-consumption, when driven by environmental and not financial reasons. Their call for more work in this area can be aligned with Campbell and Winterich (2018), who argued that in understanding various types of consumer (im)moral responses, it is important to consider individual differences in the moral domain, such as individual moral foundations, that may alter how consumers interpret the same action. Moral foundations represent the psychological systems that help people understand the difference between right or wrong (Graham et al., 2011; Haidt, 2008). Moral foundations theory (MFT) has been previously presented in relation to consumer (un)ethical behaviour (Chowdhury, 2019), consumer boycotts (Shim et al., 2018) and sustainable consumption (Kidwell et al., 2013; Watkins et al., 2016). This study will strive to complement the morally charged anti-consumption studies, and draw from MFT to examine reducing consumption for environmental reasons in the context of air travel.

Moreover, scholars have reflected on a need for more comprehensive and integrative models of environmental anti-consumption, which would include various cognitive, conative and affective antecedents of environmental anti-consumption, especially as initial research undertook a socio-cognitive approach, while forgoing other potentially relevant predictors (García-de-Frutos et al., 2018). We see opportunities to complement their findings, and in addition to the aforementioned moral factors, dip into the affective realm by examining the feelings that lead to anti-consumption. Furthermore, as environmental anti-consumption functions at the crossroads of sustainable consumer behaviour and anti-consumption in general, we can borrow from these fields to introduce different feelings that explain reducing consumption for environmental reasons. Studies have suggested that feelings of guilt (Antonetti & Maklan, 2014; Lindenmeier et al., 2017) and responsibility (Kaiser et al., 1999) are linked to prosocial consumer behaviour, but their position related to anti-consumption, although potentially relevant, remains to be determined. In explaining the decision to reduce consumption to save the environment, we will therefore introduce these two affective constructs to complement the individual moral aspects.

As the focus of previous studies was on different motivations leading to anti-consumption (Lee & Ahn, 2016), researchers have also called for more studies that would not only address the drivers/antecedents of environmental anti-consumption, but also the outcomes of this phenomenon (García-de-Frutos et al., 2018; Lee et al., 2020; Makri et al., 2020). This requires further empirical examination of the relationship between anti-consumption and more conspicuous forms of anti-consumption outcomes, such as word of mouth (WOM) and activism (Chatzidakis & Lee, 2013).

In deciding on the methodological approach, we took our cue from Makri et al. (2020), who reported that the majority of anti-consumption studies are qualitative in nature, meaning they have issues with generalizability and tend to neglect the study of relationships between manifestations of anti-consumption and their determinants. In addition, scholars who considered the particular study setting, i.e. air travel, in relation to the environment, also primarily used qualitative research techniques (Higham et al., 2014; McDonald et al., 2015). By developing and empirically testing a novel anti-consumption model in the context of air travel, this study aims to overcome some of the shortcomings outlined above and contribute to the literature on anti-consumption for environmental reasons in three primary ways. First, we suggest that moral foundations represent an underlying antecedent in explaining consumption reduction for environmental reasons. Second, we introduce feelings, i.e. guilt and personal responsibility, to complement this reasoning. Third, we test the role of positive WOM about reducing air travel and environmental activism as the outcomes of reducing consumption (i.e. air travel) for environmental reasons.

## Moral Foundations Theory

MFT is based on three underlying premises. The first premise is moral nativism and moral pluralism, which suggests that there exist multiple building blocks of moral foundations that are innate, built-in and universal. Specifically, MFT assumes that there are five moral foundations on which most cultures, as well as most individuals, build their psychological systems to decide what is right/wrong or good/bad (Graham et al., 2011; Haidt, 2008). In line with most conventional moral theories (Gilligan, 1982; Kohlberg, 1981), MFT posits that morality includes (but is not limited to) the two domains of harm/care and fairness/reciprocity. The harm/care foundation pertains to caring for others and preventing or alleviating harm. Fairness/reciprocity is concerned about issues of justice, fair treatment of individuals and proportionality. According to MFT, these two domains are called individualising moral foundations, as they emphasise individual rights and welfare. However, MFT assumes that the moral domain is usually much broader, encompassing three additional moral foundations—authority/respect, ingroup/loyalty and purity/sanctity. Ingroup/loyalty focuses on an individual’s allegiance to, and sacrifice for, their ingroups. Authority focuses on deference to hierarchical relationships, social order and traditions. Purity/sanctity emphasises self-control (and thus an abhorrence for a hedonistic, selfish lifestyle) and the importance of protecting the group from spiritual and physical contamination, such as transgressions that break social conventions. These three foundations are called binding moral foundations, as they focus on the values of groups and institutions (Graham et al., 2009).

The second premise is moral tribalism and cultural moral learning (Marcus, 2004). MFT posits that the five moral foundations are innate psychosocial systems that are universally available. However, they are modifiable and learned through cultural experiences or practices (Haidt & Joseph, 2004; Graham et al., 2009). As a result, each domain of moral foundations has varying impacts and weights across different cultures, and therefore, different moral practices are evident in different cultures. For instance, people from Western, individualistic cultures typically place greater values on individualising moral foundations than binding moral foundations. In contrast, those from Eastern cultures put equal importance on both individualising and binding moral foundations (Graham et al., 2011). Similarly, political liberals prioritise individualising moral foundations, whereas conservatives place equal weight on both. Due to moral pluralism and cultural moral learning (Haidt & Graham, 2007), individuals from different groups often have opposing views on moral issues. Ironically, polarisation is more likely to occur when everyone concerned has high moral standards and is strongly motivated to make morally right decisions (Haidt & Joseph, 2004; Graham et al., 2009).

Third, MFT assumes moral intuitionism and emotional primacy. Conventional views assume that moral judgments are based on moral reasoning or deliberative rationality (Kohlberg, 1969). Likewise, previous studies employing TPB and VBN theory have suggested that ethical decision-making is determined by beliefs and norms, such as behavioural beliefs (e.g. expectancy, outcome evaluation), perceived social norms (e.g. subjective norms) and personal values (e.g. altruistic values, egoistic values), and whether a course of action leads to valuable outcomes to the self or the greatest number of people (Oreg & Katz-Gerro, 2006; Yoon, 2011). In contrast, MFT posits that such judgments are based on innately prepared moral intuitions, often driven by gut instincts and feelings. In other words, moral judgments occur through “fast, automatic, and (usually) affect-laden processes in which an evaluative feeling of good-bad or like-dislike (about the actions or character of a person) appears in consciousness without any awareness of having gone through steps of search, weighing evidence, or inferring a conclusion” (Haidt, 2007, p. 998). As such, people have strong moral reactions (e.g. disgust, guilt) even when they have difficulty in explaining why they approve or disapprove of certain behaviours (Haidt & Joseph, 2004).

### Impact of Moral Foundations

A growing number of studies have demonstrated that MFT is a useful lens through which to understand cultural variations in morality (Graham et al., 2013), or to explain different moral judgments and values between liberal and conservative groups (Kolvera et al., 2012; Haidt & Graham, 2007; Graham et al., 2009). The findings have shown that moral foundations underlie, motivate and unite ideological positions, determining individuals’ attitudes towards a broad range of social issues, such as abortion, immigration and gay marriage (Kolvera et al., 2012). Recently, moral foundations have also been introduced to examine the evaluations of off-duty deviance in an organisational setting (Cook & Kuhn, 2020).

Several studies have applied MFT to predict consumer behaviours involving moral choices. For example, Chowdhury (2019) shows that MFT domains have distinct relationships with ethical and unethical consumption. Care/harm, fairness/reciprocity and authority/respect foundations are positively related to beliefs regarding ethical consumption. In contrast, the ingroup/loyalty foundation is negatively associated with ethical consumer actions and positively with unethical consumption. Finally, sanctity/purity is negatively associated with all forms of unethical consumer actions. Specific to green consumerism and environmental anti-consumption, several studies have shown that moral foundations can predict consumer responses to climate change (Dawson & Tyson, 2012; Dickinson et al., 2016; Jansson & Dorrepaal, 2015; Rossen et al., 2015), environmental attitudes (Feinberg & Willer, 2013) and sustainable consumption practices (Kidwell et al., 2013; Watkins et al., 2016). Kidwell et al. (2013) showed that messages which promote sustainable consumption have stronger persuasive appeals when the message appeal (emphasis on harm vs. duty) is congruent with consumers’ predispositions towards moral foundations (individualising vs. binding moral foundations). In general, harm/care and fairness have been shown to be predictive of preferences for stronger responses to climate change (Dawson & Tyson, 2012; Dickinson et al., 2016) and perceived social norms about actions on climate change (Jansson & Dorrepaal, 2015) because environmental issues have been typically recognised or framed in terms of two values: ‘harm’ to present and future generations (and to the planet), and the ‘unfairness’ of the distribution of burdens caused by climate change (Feinberg & Willer, 2011; Markowitz & Shariff, 2012). In contrast, binding moral foundations, such as loyalty (Dawson & Tyson, 2012) and authority (Jansson & Dorrepaal, 2015), have a negative association with support for actions on climate change. Those who place a greater weight on binding moral foundations prioritise stability, social order and tradition (Graham et al., 2009), which may suppress moral concerns about harm reduction and fairness (Haidt & Graham, 2007). Overall, the findings demonstrate that MFT is a useful means to assess the role of moral foundations in guiding anti-consumption behaviours related to environmental issues.

## Hypotheses

### Moral Foundations

The literature reviewed above suggests that moral foundations can predict anti-consumption behaviour. Anti-consumption involves a trade-off between concerns about harm/fairness and a preference for social order, tradition and the free market. As such, individualising and binding moral foundations may be of particular interest to understand the effect of morality on anti-consumption for environmental reasons. The limited research outlined earlier suggests that individualising moral foundations are likely to be positively associated with responses to climate change (Dickinson et al., 2016) and prosocial behaviour (Clark et al., 2017). As individualising moral foundations prioritise harm reduction/fairness, they are likely to endorse actions on climate change and environmental degradation to prevent harm and unfairness to present and future generations (Koleva et al., 2012). Based on these premises, we suspect that people who are more sensitive to individualising moral foundations will be more likely to curb their consumption to help the environment, meaning they will reduce their air travel.

#### Hypothesis 1a

Individualising moral foundations positively influence intentions to reduce consumption for environmental reasons.

In contrast, binding moral foundations are associated with the desire to maintain social order and traditional social structures, suppressing consumer actions, such as boycott intentions (Shim et al., 2018). It is suggested that binding moral foundations inhibit boycott intentions in pursuit of harmonious orchestration of the whole community when the entity (e.g. company) is perceived as a valid and active community member. Previous studies have suggested that environmental issues largely fail to activate the moral intuitions held by those who are politically right-wing and have a high degree of binding moral foundations (Feinberg & Willer, 2013). Three domains of binding moral foundations have a negative association with consumer responses to climate change or environmental activism (Dawson & Tyson, 2012; Jansson & Dorrepaal, 2015). Hence, we predict that individuals with high binding moral foundations will be less willing to reduce their consumption for environmental reasons:

#### Hypothesis 1b

Binding moral foundations negatively influence intentions to reduce consumption for environmental reasons.

### Personal Responsibility

In addition to moral foundations, which constitute the main theoretical framework of this study, personal responsibility is also included in the analysis. Several researchers have noted that it is an under-researched topic within marketing and management and, therefore, calls for more research (e.g. Luchs et al., 2015). Wells et al. (2011) in particular pointed out that responsibility has been neglected in studies of pro-environmental consumer behaviour. The concept of personal responsibility is formulated in different ways across academic studies. For example, Luchs and Miller (2015) explained consumer responsibility for sustainable consumption in terms of prosocial and pro-environmental values. Some scholars define personal responsibility as responsibility for the environment (e.g. Wells et al., 2011) or environmental damage (Wu & Yang, 2018). We define personal responsibility following Kaiser and Shimoda’s (1999) conceptualisation of feelings of responsibility, described as an individual’s feeling of personal obligation towards the environment, as also used in previous studies (e.g. Bamberg & Möser, 2007; Verma et al., 2019).

To activate moral behaviours, an individual must ascribe personal responsibility for the issue at hand (Schwartz, 1977). Though the relationship between moral foundations and ascribing responsibility has not been empirically tested, the related literature suggests that moral foundations should be related to personal responsibility. Individualising moral foundations, such as fairness and harm/care, focus on the values of fairness/reciprocity and sympathy/empathy/care. To maintain fair relationships, both parties must accept personal responsibility and the norms of reciprocity. Individualising moral foundations are also associated with responsibility for taking care and doing no harm. On the other hand, binding moral responsibility is based on group orientation, obligation and compliance (Kidwell et al., 2013), which means it could ascribe responsibility to a group and inhibit the attribution of personal responsibility. Hence, we predict that:

#### Hypothesis 2a

Individualising moral foundations positively influence personal responsibility.

#### Hypothesis 2b

Binding moral foundations negatively influence personal responsibility.

The next hypotheses refer to the relationship between personal responsibility and its consequences. There are numerous studies that attest to the significance of a sense of responsibility as a predictor of behaviour (e. g. Ernst et al., 2017; Kaiser et al., 1999; Yu et al., 2017). For example, scholars who based their research on VBN theory confirmed that ascription of responsibility which reflects the obligation to act in a certain manner influences anti-consumption behaviour (Jakocevic & Steg, 2013; Unal et al. 201). In contrast, our conceptualisation of responsibility focuses on the obligation towards the environment, which may influence the way people modify their behaviour in order to save it. According to Cherrier (2009), consumers’ sense of environmental responsibility increases their concern for the environment, which then leads to stronger behavioural intentions to act sustainably. Furthermore, Kaiser and Shimoda (1999) demonstrate that how responsible a person feels for the environment is a significant predictor of ecological behaviour. Hence, we posit that individuals who feel a personal obligation towards the environment will be more likely to reduce consumption for environmental reasons:

#### Hypothesis 3a

Personal responsibility positively influences intentions to reduce consumption for environmental reasons.

Another hypothesised consequence of personal responsibility is feelings of guilt. The existing literature offers ample evidence that the perception of responsibility is an important driver of feelings of guilt. For example, Wicker et al. (1983) found that guilt follows from feelings of responsibility. McGraw (1987) experimentally confirmed that guilt is a linear function of responsibility, as long as responsibility is conceptualised as self-blame and not as self-attributed causality. Self-blame refers to the moral condemnation of one’s own actions, which is similar to our concept of personal responsibility. In the context of fair-trade consumption, Lindenmeier et al. (2017) also pointed out that perceptions of responsibility are an antecedent of guilt. Although these are different settings, we believe that the relationship will be similar when examining guilt in relation to environmentally harmful behaviours, such as air travel. The more people feel responsible for the state of the environment, the more guilt they expect to feel for the impact their consumption (i.e. air travel) has on the planet. As such, we posit:

#### Hypothesis 3b

Personal responsibility positively influences feelings of anticipated guilt.

Personal responsibility may also lead to another outcome in our model, i.e. environmental activism, which can be defined as “intentional and conscious civic behaviours that are focussed on systemic causes of environmental problems and the promotion of environmental sustainability through collective efforts” (Alisat & Riemer, 2015, p. 14). When individuals believe they have a responsibility to make a change, this leads them to bond with other community members in an effort to improve society (Hollenbeck & Zinkhan, 2006). Similarly, Paco and Gouveia Rodrigues (2016) suggested that personal responsibility does not only influence individual consumer behaviour, but also their involvement in social movements, such as environmental activism. They found empirical support for a positive relationship between responsibility and activism. Based on this, we hypothesise:

#### Hypothesis 3c

Personal responsibility positively influences environmental activism.

### Feelings of Guilt

Although studies on consumer behaviour have captured a range of emotions, such as love, anger, fear, guilt and worry (Burnett & Lunsford, 1994), we focus on guilt because it has been described as a consumer state that precedes an action (or inaction) that has consequences for the wider society (Theotokis & Manganari, 2015). Guilt can be defined “as a negative emotion which results from a consumer decision that violates one′s values or norms” that leads to compliant or altruistic behaviour (Burnett & Lunsford, 1994). In understanding emotions, such as feelings of guilt, we need to distinguish between current or past emotional experiences that influence behaviour and anticipated emotions that may affect behaviour. If consumers anticipate the feelings of guilt they would experience if they engaged in a problematic behaviour, then their behaviour is guided by this anticipatory affective experience (Steenhaut & Van Kenhove, 2006). In this study, we are interested in anticipated guilt, i.e. an anticipated negative emotion that arises from travelling by air.

We develop the next hypothesis based on one of the key assumptions of MFT, i.e. moral intuitionism and emotional primacy. The MFT assumes that moral choices are based on affective, automatic and intuitive processing (Haidt & Graham, 2007). Therefore, we predict that immediate, intuitive and spontaneous moral responses, such as feelings and gut reactions, play an important role in motivating morally relevant actions. In the context of anti-consumption, guilt is a salient emotional response that influences consumer choices (McDonald et al., 2015; Mkono & Hughes, 2020). Guilt is a “self-conscious moral emotion” (Haidt, 2003). Individuals with high individualising moral foundations are more likely to feel guilty when they perceive that they are the perpetrator of unfair treatment of others, including future generations and even non-human characters in video games (Grizzard et al., 2014; Weaver & Lewis, 2012). Intuitions about social order and stability can often suppress moral intuitions and concerns, such as guilt associated with unfair treatment of other individuals (Haidt & Graham, 2007, 2009). Accordingly, individuals with high binding moral foundations may feel less guilty for engaging in problematic behaviour, such as air travel. Hence, we posit:

#### Hypothesis 4a

Individualising moral foundations positively influence feelings of anticipated guilt.

#### Hypothesis 4b

Binding moral foundations negatively influence feelings of anticipated guilt.

We are also interested in the potential consequences of feelings of anticipated guilt. Specifically, in Hypothesis 5a our aim is to test the relationship between feelings of anticipated guilt and intentions to reduce consumption (i.e. air travel) for environmental reasons. Previously, Antonetti and Maklan (2014) investigated the relationship between the guilt felt after an unethical purchase and the future intention to engage in sustainable consumption. However, their findings on the relationship between guilt and behavioural intention were rather inconclusive, as they found a significant positive effect of guilt on intention in one study, but not in the other. At the group level of consumer behaviour, Harth et al. (2013) confirmed that a positive effect of guilt on the intention to repair environmental damage, but not on the intentions to punish environmental “sinners” or favour environmental protection. On the other hand, Wang and Wu (2016) found that feelings of guilt lead to a stronger intention across two studies: one conceptualising intention with respect to resistance to non-energy conserving household appliances, and another with respect to purchasing energy conserving household appliances. We believe that those individuals who anticipate feeling guilty as a result of a particular environmentally harmful behaviour (i.e. air travel), will be more prone to reducing this behaviour to save the environment. Therefore, the following hypothesis is proposed:

#### Hypothesis 5a

Feelings of anticipated guilt positively influence intentions to reduce consumption for environmental reasons.

The next hypothesis argues that feelings of guilt have a positive influence on WOM about reducing air travel. The relationship between emotions and the WOM intention has already been studied in the literature, but with varying degrees of support and in different contexts. White (2010), who focussed on service encounters, reported that negative emotions (captured by guilt, humiliation and depression) are not a significant predictor of positive WOM intention. In contrast, Soscia (2007) found a significant effect of feelings of guilt on negative WOM. Similarly, Lindenmeier et al. (2017) pointed to a positive impact of guilt on fair-trade buying intentions, the latter being composed of several items, including positive WOM. Given that Lindenmeier et al.’s (2007) study considers prosocial consumer decisions, similar to our study, we believe their logic could be transferred to our study setting and explain positive WOM about reducing the consumption of air travel. Analogously, individuals who anticipate strong feelings of guilt associated with problematic behaviours may try to do everything possible to reduce such behaviours, and not only their own, but also those of others. Hence, they will actively endorse reducing consumption through WOM. Based on these outlines, we develop the following hypothesis:

#### Hypothesis 5b

Feelings of flight guilt positively influence WOM about reducing consumption (i.e. air travel).

### Outcomes of Reducing Consumption for Environmental Reasons

#### WOM

We now turn to examine two potential consequences of consumers’ intentions to reduce consumption for environmental reasons, starting with WOM about reducing air travel and followed by environmental activism. The existing literature is replete with empirical evidence for a significant relationship between different types of behavioural intentions and WOM, especially in the context of tourism. For example, Agag and El-Masry (2016) demonstrated that the behavioural intention to participate in an online travel community is associated with positive WOM communication. Similarly, a study in the service context showed that different types of commitment (similar to behavioural intention) influence positive WOM intentions (Fullerton, 2011). Furthermore, Liu and Lee (2016) found that customers’ WOM communication is positively associated with future behavioural intentions to revisit a low-cost airline. It is our contention that intention to spread positive WOM about reducing consumption is reinforced by one’s intention to reduce consumption to save the environment, as stated in the following hypothesis.

##### Hypothesis 6

Intentions to reduce consumption for environmental reasons positively influence WOM about reducing consumption (i.e. air travel).

#### Environmental Activism

Finally, in the last hypothesis we posit that intentions to reduce consumption for environmental reasons lead to greater engagement in environmental activism. Although Stern (2000) described environmental activism as one of the four types of pro-environmental behaviour, he also suggested that this concept differs considerably in terms of the impact and intent of environmental protection. In general, previous studies indicated that variables of environmental behaviour are relatively poor predictors of environmental activism (e.g. Seguin et al., 1998). Bearing this in mind, Dono et al. (2010) reported mixed support for the influence of different types of pro-environmental behaviour on environmental activism, suggesting the latter depends on the type of behaviour. Many consumption practices, even air travel, are rationalised as being ‘necessary’ or ‘unavoidable’ (Gossling et al., 2019), which means limiting this behaviour is a difficult choice. Therefore, for individuals that do make the decision to reduce consumption for environmental reasons it may represent a stepping-stone to becoming more active citizens and joining the environmental movement. Hence, we propose that positive intentions to reduce consumption of air travel for environmental reasons may encourage individuals to collectively and more actively participate in efforts to save the environment through environmental activism. As such, we propose:

##### Hypothesis 7

Intentions to reduce consumption for environmental reasons positively influence environmental activism.

**Fig. 1:**
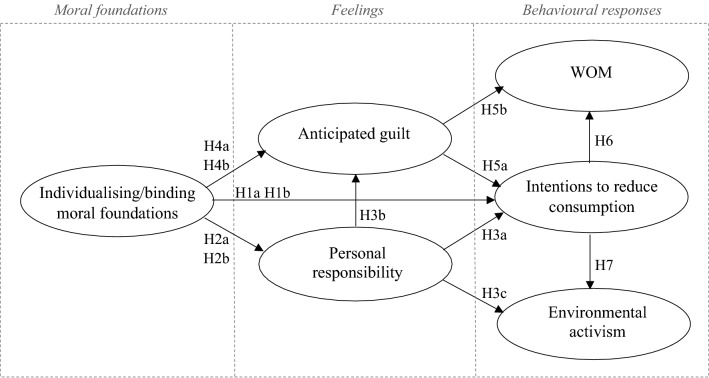
Conceptual model

In order to visually present the proposed hypothesised relationships, we developed the conceptual model shown in Fig. 1.

## Methodology

### Sample

A cross-sectional survey was conducted to test the proposed hypotheses. We used Prolific, a professional online panel, and randomly recruited 579 respondents from the UK, who were compensated at the equivalent of £8 per hour. To improve data quality, we included a directed query attention check (“Please tick the answer ‘Somewhat agree’”, see Abbey & Meloy, 2017), which excluded 68 inattentive respondents who failed to tick the requested answer. This resulted in a final sample of 511 respondents (71.6% female, median age 35 years, 17% 18–24, 35% 25–34, 24% 35–44 and 24% 45+). In terms of education, 37% had completed secondary school, 43% a university undergraduate programme and 20% post-graduate programme or doctoral degree programme. The majority (46%) were employed full time and 24% part-time, with 15% unemployed, 4% retired and 9% students. The countries of residence were England (86%), Scotland (7%), 6% Wales and 1% Northern Ireland. 55% Evaluated their household income as average, 28% below and 18% above average.

Several procedural measures were included in order to minimise common method bias (MacKenzie & Podsakoff, 2012). In order to increase the ability of respondents to respond accurately, we pretested the questionnaire to ensure questions were comprehensible. We informed respondents that the survey would be used for academic research purposes, which could increase their motivation to respond accurately. The research was conducted using a computer-assisted questionnaire, thus minimising the effects of interviewer presence and the potential social desirability of answers. The data were collected in January 2020, before the start of the COVID-19 pandemic.

### Measures

All constructs were measured with established scales. Some of the scales were adapted to the chosen context, i.e. air travel. **Moral foundations** were measured with 20 items, using the shortened Moral Foundations Questionnaire (MFQ20) from Graham et al. (2008) which includes items related to Harm, Fairness, Ingroup, Authority and Purity. In line with the mapping of the moral foundations (Graham et al., 2011), Harm and Fairness were assigned to **individualising moral foundations**, whereas **binding moral foundations,** binding people together into larger groups and institutions, incorporated Ingroup, Authority, and Purity moral foundations (see Table 1). **Anticipated guilt** was measured by adapting the scale from Lindenmeier et al. (2017), which uses four items, including: “I would feel guilty for the impact my air travel has on the planet”. **Personal responsibility** was measured with the scale from Kaiser et al. (1999) which has four items, including: “I feel at least co-responsible for the presently occurring environmental problems” (1 = completely disagree, 5 = completely agree). **Intention to reduce consumption (i.e. air travel) for environmental reasons** included three items by adapting the scale from Grau and Folse (2007). A sample items is: “I would be willing to limit my air travel to reduce my carbon footprint.” **WOM** was measured with three items (Hofenk et al., 2019), including: “I would say positive things about limiting air travel to other people.” **Environmental activism,** measured as in Dono et al. (2010), included five items, such as: “I participate in events organised by environmental groups.”

**Table 1:** Items, loadings and reliabilities

Scale (Cronbach’s *α*)/items	Standard factor loading	*t*-test	Standard error
Individualising moral foundations (0.770)			
Fairness/Cheating, e.g. Justice is the most important requirement for a society	0.652	–	
Care/Harm, e.g. Compassion for those who are suffering is the most crucial virtue	0.939	9.092	0.177
Binding moral foundations (0.831)			
Ingroup/loyalty, e.g. I am proud of my country’s history	0.756	–	
Authority/Subversion, e.g. Respect for authority is something all children need to learn	0.919	16.598	0.047
Purity/sanctity, e.g. People should not do things that are disgusting, even if no one is harmed	0.697	15.455	0.060
Personal responsibility (0.854)			
(R) Because my personal contribution is very small, I do not feel responsible for air pollution	0.784	–	
(R) I do not feel responsible for the greenhouse effect	0.838	18.917	0.055
I feel responsible for the condition of the air	0.751	17.00	0.051
I feel at least co-responsible for the presently occurring environmental problems	0.715	16.109	0.048
Anticipated guilt (0.920)			
I would feel guilty for the impact my air travel has on the planet	0.780	–	
I would blame myself for the impact my air travel has on the planet	0.790	28.705	0.035
I would feel ashamed for harming the planet with my air travel	0.810	29.226	0.039
I would feel irresponsible for harming the planet with my air travel	0.600	22.114	0.039
Intentions to reduce consumption for environmental reasons (0.946)			
I would be willing to limit my air travel to reduce my carbon footprint	0.956	–	
I would consider limiting my air travel to reduce my carbon footprint	0.945	44.240	0.022
It is likely that I would limit my air travel to reduce my carbon footprint	0.880	34.686	0.028
WOM about reducing air travel (0.921)			
I would say positive things about limiting air travel to other people	0.833	–	
I would recommend limiting air travel to people who seek my advice	0.937	27.729	0.044
I would encourage friends and relatives to limit air travel	0.911	26.628	0.047
Environmental activism (0.855)			
I participate in events organised by environmental groups	0.809	–	
I give financial support to an environmental group	0.732	17.043	0.059
I circulate petitions demanding an improvement of government policies regarding the environment	0.721	16.702	0.065
I participate in protests against current environmental conditions	0.773	18.186	0.049
I write letters to firms that manufacture harmful products	0.698	16.140	0.042

To reduce potential common method bias, we relied on established measures, made an effort to keep the measurement of dependent and independent variables as distant as possible and used various rating scale formats for different constructs (Podsakoff, MacKenzie, & Podsakoff, 2012). All scales’ items, their loadings and reliabilities are shown in Table 1.

## Results

### Measurement Model

We tested the cross-sectional dataset with the marker variable technique for potential effects of common method bias (Bagozzi, 2011). A response to the question “How many cars are there in your household?” was selected as a marker variable, because it is theoretically not related to the constructs in our conceptual model. The addition of a marker variable had no effect on the fit of the measurement model or factor loadings of indicators on substantive latent variables. Moreover, three out of six correlations between the marker variable and the latent variables were not statistically significant and the significant coefficients were small in magnitude (*r* < 0.14). This leads us to the conclusion that common method bias is unlikely to influence our results.

We performed SEM using LISREL 8.80 to evaluate the measurement properties of the operationalisations of our focal constructs and test the research hypotheses. We estimated a measurement model that fitted well with the data (*χ*^2^ = 85.125; df = 31; CFI = 0.976; RMSEA = 0.056; SRMR = 0.02). Examination of the composite reliabilities and AVEs, reported in Table 2, provides support for convergent validity.

**Table 2:** Validity matrix

Construct	CR	AVE	1	2	3	4	5	6	7
Individualising moral foundations (1)	0.78	0.65	**0.808**						
Binding moral foundations (2)	0.84	0.65	0.112*	**0.796**					
Personal responsibility (3)	0.86	0.60	0.340***	− 0.043	**0.773**				
Anticipated guilt (4)	0.92	0.75	0.324***	− 0.106*	0.505***	**0.866**			
Intentions to reduce consumption (5)	0.95	0.86	0.374***	− 0.069	0.380***	0.602***	**0.927**		
WOM (6)	0.92	0.80	0.314***	− 0.073	0.380***	0.658***	0.756***	**0.894**	
Environmental activism (7)	0.86	0.56	0.181***	− 0.028	0.350***	0.281***	0.353***	0.288***	**0.748**

Square root of AVE in bold on the diagonal

Significance of correlation coefficients (below the diagonal): *p < 0.05; **p < 0.01; ***p < 0.001

### Structural Model

Consistent with our research hypotheses, we estimated a model that includes the paths from individualising and binding moral foundations to personal responsibility, anticipated guilt and intentions to reduce consumption (i.e. air travel) for environmental reasons, followed by WOM about reducing air travel and environmental activism as the main outcomes. We also included age, gender, income and number of flights in the previous period (before January 2020) as control variables in the model. We fitted the structural model and obtained an acceptable fit (*χ*^2^ = 153.489; df = 54; CFI = 0.957; RMSEA = 0.059; SRMR = 0.033). The model accounts for more than 42% of the variance in intentions to reduce consumption, 64% of variance in WOM and 17.8% of variance in environmental activism. Moreover, the estimates of the path coefficients (Table 3) are all significant with two exceptions (binding moral foundations have no direct significant effect on intentions to reduce consumption, and personal responsibility has no direct significant effect on these intentions). Regarding the control variables, age has a positive impact on intentions, while income has a negative one. The number of flights in the previous period has no significant impact on intentions, and neither does gender.

**Table 3:** Structural model results

Relationship	Nature of the relationship	Standard path coefficient	*t*-test	Standard error
Individualising moral foundations → Intentions to reduce consumption	H1a	0.203***	4.453	0.148
Binding moral foundations → Intentions to reduce consumption	H1b	− 0.038	− 0.961	0.074
Individualising moral foundations → Personal responsibility	H2a	0.349***	6.715	0.118
Binding moral foundations → Personal responsibility	H2b	− 0.082*	− 1.681	0.063
Personal responsibility → Intentions to reduce consumption	H3a	0.068	1.403	0.069
Personal responsibility → Anticipated guilt	H3b	0.437***	9.152	0.066
Personal responsibility → Environmental activism	H3c	0.251***	4.891	0.058
Individualising moral foundations → Anticipated guilt	H4a	0.188***	3.939	0.151
Binding moral foundations → Anticipated guilt	H4b	− 0.108***	− 2.484	0.079
Anticipated guilt → Intentions to reduce consumption	H5a	0.502***	10.755	0.048
Anticipated guilt → WOM	H5b	0.318***	7.696	0.039
Intentions to reduce consumption → WOM	H6	0.565***	13.868	0.037
Intentions to reduce consumption → Environmental activism	H7	0.255***	5.218	0.038
Age → Intentions to reduce consumption	Control	0.072*	1.945	0.034
Gender → Intentions to reduce consumption	Control	− 0.050	− 1.189	0.110
Income → Intentions to reduce consumption	Control	− 0.084**	− 2.184	0.067
No. of flights → Intentions to reduce consumption	Control	0.054	1.441	0.037

Significance of path coefficients: *p < 0.05; **p < 0.01; ***p < 0.001

Importantly, the results of the structural model provide support for the majority of our hypotheses. The effects of individualising (H1a) moral foundations on intentions to reduce consumption for environmental reasons are significant. Individualising moral foundations also positively influence personal responsibility (H2a) and anticipated guilt (H4a), while the influence of binding moral foundations on responsibility (H2b) and guilt (H4b) is negative. Additionally, personal responsibility significantly and positively impacts anticipated guilt (H3b) and environmental activism (H3c). Flight guilt in turn has a positive and strong influence on intentions (H5a) and WOM (H5b). Intention to reduce consumption positively and significantly influences both WOM (H6) and environmental activism (H7). However, we could not find support for the hypotheses linking binding moral foundations (H1b) and personal responsibility (H3a) with intentions to reduce consumption (i.e. air travel) for environmental reasons.

### Mediation Analysis

Additionally, we tested (1) whether individualising and binding moral foundations influence intentions to reduce consumption by increasing guilt, which in turn affects these intentions, and (2) whether individualising and binding moral foundations influence intentions to reduce consumption by increasing personal responsibility, which in turn affects the intentions.

We used a serial multiple mediator model (PROCESS Model 6, Hayes 2018), in which personal responsibility and guilt are two mediators that influence each another. Individualising and binding moral foundations serve each other as covariates. The total effect of individualising and binding moral foundations on intentions to reduce consumption for environmental reasons is the sum of the direct effect, specific indirect effects and serial specific indirect effects of the two serial mediators, personal responsibility and guilt. We bootstrapped 5000 samples to construct 95% confidence interval tests for indirect effects.

The results show that individualising moral foundations increased intentions to reduce consumption for environmental reasons:

- serially through personal responsibility and guilt (0.116, 95% CI = 0.069 to 0.176),
- through guilt independent of personal responsibility (0.181, 95% CI = 0.082 to 0.292), and
- not through personal responsibility independent of guilt (0.049, 95% CI =  − 0.004 to 0.113).

There is also a significant direct effect of individualising moral foundations on intentions to reduce consumption (0.43, 95% CI = 0.2583 to 0.6018).

The results for binding moral foundations show lower intentions to reduce consumption only through guilt independent of personal responsibility (− 0.073, 95% CI =  − 0.144 to − 0.006) and neither serially (− 0.025, 95% CI =  − 0.057 to 0.004) nor through personal responsibility independent of guilt (− 0.011, 95% CI =  − 0.033 to 0.003). There is no direct effect of binding moral foundations on intentions to reduce consumption for environmental reasons (− 0.055, 95% CI =  − 0.173 to 0.064).

## Discussion

This study examined the antecedents and consequences at the intersection of sustainable consumption and anti-consumption in the context of reducing air travel for environmental reasons, and provided a novel framework for understanding this phenomenon. We have attempted to provide empirical evidence on how a conjunction of moral and affective determinants may interact to influence consumers’ intentions to reduce consumption to help the environment, WOM about reducing consumption (i.e. air travel) and environmental activism. In doing so, we aim to advance the attitude-related theories, such as the TPB and VBN theory. By moving away from the normative/reasoned based approach into the affective and moral realms, we contribute to the research on anti-consumption for environmental reasons in the following ways.

Our first contribution lies in introducing MFT not only as a driver of consumption reduction for environmental reasons, but also of feelings of anticipated guilt and responsibility. Our results therefore contribute to the conversation on sustainable consumption and anti-consumption in relation to consumer ethics, which has hitherto been principally conceptual in nature (e.g. Azevedo, 2020; Garcia-Ruiz & Rodriguez-Lluesma, 2014), while empirical studies have reported mixed support for the influence of ethical ideology on anti-consumption (e.g. Sadbury-Riley et al. 2018; Peifer et al., 2020) or sustainable/prosocial consumption (Zou & Chan, 2019; Chowdhury, 2018). With the introduction of MFT, we supplement the findings of these earlier studies with an alternative morality-related concept. Accordingly, by employing a set of moral intuitions, we move beyond attitudes and norms, as the main predictors found in TPB (e.g. Chen, 2016), to determine how moral foundations predict feelings and behavioural responses. In contrast to VBN theory, which captures the effects of general values on behaviour and responsibility (Jakocevic & Steg, 2013; Unal et al. 2019), moral foundations represent a sub-set of wider values research that specifically focuses on morality (Watkins et al., 2016).

In our study, we have found partial support for the proposition that morality encourages consumers to reduce consumption in the future, which expands the findings of Chowdhury (2019) who also partially confirmed the link between moral foundations and ethical beliefs in relation to prosocial actions. We found that only individualising moral foundations positively affected intentions to reduce consumption, while the influence of binding moral foundations was non-significant. The findings echo those by Shim et al. (2018), where individualising moral foundations positively influenced boycott intention and binding moral foundations had no influence for the South Korean and Singaporean samples. While we expected that binding moral foundations would dissuade individuals from reducing consumption, no direct influence was found, suggesting consumers do not base this decision on social and traditional aspects, but rather rely on their perceptions of fairness and welfare.

Further looking into the role of MFT in explaining feelings, the findings suggest the importance of distinguishing between individualising and binding moral foundations, with the former displaying a positive and the latter a negative effect on the feelings of guilt and responsibility. We therefore extend the literature employing VBN theory, where general values predict personal norms that represent the feelings of moral obligation *to act in a certain way* (Schwartz, 1977). We focussed instead on how moral intuitions, which have a narrower focus on morality compared to values (Graham et al., 2009), influence other potentially relevant, but under-researched affective concepts, i.e. personal responsibility, which represents the feeling of personal obligation *towards the environment*, and guilt which represents the negative feeling associated with *performing a particular behaviour.* Although consistent with previous MFT literature, which found the influence of different moral foundations going in opposite directions in the study of political action and rhetoric (Haidt & Graham, 2007) and consumer ethics (Chowdhury, 2019), the unique aspect of our study is the proposed feelings as an outcome of MFT.

Our second contribution refers to establishing the role of feelings in understanding diverse behavioural outcomes, i.e. the reduction of consumption for environmental reasons, WOM about reducing consumption (i.e. air travel) and environmental activism. To begin with, the interplay of responsibility, anticipated guilt and intentions to reduce consumption was interesting, and this contributes to the anti-consumption literature which has tended to focus on the socio-cognitive perspective (see García-de-Frutos et al., 2018; Yarimoglu et al., 2019; Zhang et al., 2019) rather than on the affective perspective (e.g. Boujbel & d’Asouts, 2015). Our study showed how important the feeling of guilt individuals anticipate is in reducing consumption. Not only that, guilt also mediates the relationship between moral foundations and reducing consumption, thus confirming the proposition of earlier studies in other settings in which guilt was reported as a significant mediator between ethical perceptions and intentions (Lindenmeier et al., 2017; Steenhaut & Van Kenhove, 2006). Moreover, guilt also predicted WOM, a finding which contributes to the previous literature that focussed primarily on guilt in relation to intentions and/or behaviours (Antonetti & Maklan, 2014; Onwezen et al., 2013), but not on the outcomes that follow. Guilt in the context of the current study seems to be such a strong and profound feeling that it urges people not only to change their own behaviour, but also makes them want to talk to their peers to encourage them to engage in less consumption (i.e. air travel). In addition, personal responsibility significantly influenced feelings of guilt and activism. The more people felt responsible for the planet, the more the impact of air travel made them feel guilty and the more involved they became in environmental movements. Interestingly, although Kaiser et al. (1999) found responsibility to be the main predictor (coupled with knowledge and values) of ecological behavioural intentions, we did not find support for its direct influence on intentions to reduce consumption for environmental reasons, but the influence was indirect through guilt.

The third theoretical contribution of this study is made by highlighting some of the consequences of anti-consumption behaviour. More specifically, our study shows that the intention to reduce consumption leads to WOM about reducing consumption (i.e. air travel) and environmental activism, confirming the notion of Lee et al. (2020) that anti-consumption can take on more conspicuous forms. This finding allows us to contribute to the literature on anti-consumption, since it shows that this decision has consequences that are directed at others, either by suggesting that they do the same through WOM, or by actively engaging in behaviours that become visible to others in the form of environmental activism.

### Practical Implications

In recent years, efforts have been made to promote anti-consumption as a means to achieve sustainability goals (García-de-Frutos et al., 2018; Lee et al., 2020) by governments, public policy-makers, activist groups and non-profit organisations. The present research findings highlight three areas that may prove effective in curbing consumption, i.e. changing an individual’s air travel intentions: an individual’s moral foundations, their perception of personal responsibility for environmental problems and their perception of guilt.

First, organisations may need to appeal to individuals’ morality to get them to think about reducing consumption for environmental reasons. We conjecture that endorsing individualising moral foundations may be a more efficient strategy than attempting to discourage binding moral foundations. In doing so, we rely on results showing that individualising moral foundations increase intention to reduce consumption for environmental reasons, as well as feelings of guilt and personal responsibility, whereas binding moral foundations reduce guilt and responsibility, but do not affect intention. Thus, it is critical to match communication with audiences that differ in terms of their prevailing moral foundations (individualising vs. binding). Indeed, existing literature indicates idiosyncratic differences among individuals in their sensitivity to moral foundations or moral norms (Cook & Kuhn, 2020). To illustrate, for consumers with a high sensitivity to individualising moral foundations (as expressed, for example, in their high sensitivity to cheating or reducing equality), it is important to clearly communicate how governments, NGOs and other relevant parties respect and care about individual customers. For example, designing marketing campaigns that address the harm and fairness of moral issues related to environmentally friendly consumption may not only prompt consumers with stronger individualising foundations to form behavioural responses (reduction of consumption and environmental activism), but also increase their feelings of responsibility and guilt. Moreover, previous research has demonstrated that an abstract (as opposed to concrete) mindset increases individualising values and decreases concerns about binding values (Napier & Luguri, 2013). This finding could add to our recommendation in the sense that messages of governments or NGOs should be abstract enough to promote individualising and discourage binding moral foundations. These two layers of foundations in turn will have a reverse effect on subsequent feelings of responsibility, guilt and intention to reduce consumption for environmental reasons.

Second, another area that should be considered is the individual’s feeling of anticipated guilt. Given its prominent role in our conceptual model, it is important to suggest how to manage this feeling. One possibility is the design of advertising appeals that highlight emotions related to concern for the environment. More specifically, the use of the so-called guilt-inducing nudges (Eyal, 2014) could lead observers to experience higher levels of guilt. Moreover, this finding implicitly provides support for the flight-shaming movement, as its success may be due to boosting this feeling.

Third, due to the role of the feeling of personal responsibility, this could be deliberately encouraged among consumers to raise their intentions (through guilt) and thus foster activism. It is expected that communication appeals reminding consumers of their own responsibility will work towards this goal. However, it is important to develop communication strategies carefully, because there are some limitations with respect to focussing on personal responsibility. One limitation is that too much emphasis on personal responsibility could lead to consumers rebelling against taking this on. For this reason, Luchs et al. (2015) suggest that these efforts should also focus on other stakeholders, not just consumers. Nonetheless, the involvement of multiple stakeholders could inhibit an individual's responsibility, since a person is less likely to take responsibility for their own actions if others are also perceived to be accountable (Wu & Yang, 2018). Therefore, communication should emphasise personal responsibility to a moderate extent and not involve too many stakeholders. Another solution to resolve this controversy is to create communication strategies aimed at smaller groups of consumers, as it has been shown that the likelihood of diffusion of responsibility increases with group size (Przepiorka & Diekmann, 2018). With this in mind, designing the most personalised type of communication possible would be the most effective. Raising personal responsibility in this context could have broader societal implications, as it can encourage individuals to play a more active role by joining the environmental movement through established global or local NGOs, or even to start their own campaigns in the fight against climate change.

### Limitations and Future Research Opportunities

There are some limitations to this study that need to be addressed. First and foremost, the study was conducted just before the outbreak of COVID-19, which brought the air travel industry to a standstill and from which it will take some time to recover (OECD, 2020). If the study was carried out during the COVID-19 crisis, we would also have to take this perspective into account, since limiting air travel in this context is not due to environmental factors, but to wider societal changes.

The chosen study setting is very important with regard to reducing carbon emissions in general, and the carbon footprints of individuals in particular. However, the generalisability of the results to other problematic industries, such as construction and food, may be limited, as compared to these flying is often perceived as a luxury that people are more willing (and able) to give up (Moran et al., 2020; Kantenbacher et al., 2019; Thøgersen, 2021). On the other hand, considering the recent study by the EIB (2021), there may be some potential in adapting the proposed model and testing it for other consumer behaviours that could contribute to solving the problem of climate change, such as reducing electricity use, limiting waste production and the substitution of other foods for meat (Thøgersen, 2021).

Another limitation relates to the use of the crowdsourced panel Prolific to collect the data. More specifically, there is the problem of representativeness: Prolific samples tend to have a slight female bias, with a younger and more highly educated population (Prolific, 2018). Other issues include self-selection, as participants self-select for studies and can stop responding at any time, and nonnaiveté, as participants gain experience with social science studies over time (Goodman & Paolacci, 2017).

In terms of our conceptualisations, the concept of moral foundations has evolved. For instance, researchers have proposed a candidate for a sixth dimension of moral foundations, named liberty/oppression, which focuses on concerns about domination and coercion (Iyer et al., 2012). Recent MFT studies have also proposed a new concept called moral progressivism, which refers to the degree to which an individual endorses individualising moral foundations over binding moral foundations (Clark et al., 2017). Future research can examine such novel conceptualisations of moral foundations and explore their relationships with anti-consumption. Furthermore, to make valid inferences about the causality of relationships between constructs, experimental approaches could be employed. For example, appeals consistent with individualising and binding moral foundations could be designed to manipulate an individual’s levels of moral foundations and measure subsequent intentions or behaviours (similar to the work done by Kidwell et al., 2013).

Finally, we focussed on specific moral and affective factors that determine environmental anti-consumption. The inclusion of other emotions, such as anger, pride (Antonetti & Maklan, 2014; Antonetti et al., 2020) or shame as an integral part of flight-shaming (leading to reduced air travel) (Claeys, 2020), could be beneficial, as could other cognitive factors that would explain reducing consumption for environmental reasons from a different perspective.

## Footnotes

1 Following Leary et al. (2014), sustainable consumption is defined as behaviour intended to meet the needs of the current generation and to *benefit the environment* without jeopardising the ability of future generations to satisfy their needs.
